# A covalent recognition strategy enables conspecific mate identification

**DOI:** 10.1101/2025.05.30.657074

**Published:** 2025-06-03

**Authors:** Sukjin S. Jang, Sanjana Mandala, Hanjie Jiang, Xiao Zhang, Phillip A. Cole, Josefina del Mármol

**Affiliations:** 1 Department of Biological Chemistry and Molecular Pharmacology, Harvard Medical School, Boston, MA, US; 2 Division of Genetics, Department of Medicine, Brigham and Women’s Hospital, Boston, MA, US; 3 Department of Chemistry, Massachusetts Institute of Technology, Cambridge, MA, US; 4 Howard Hughes Medical Institute, Boston, MA, US

## Abstract

The olfactory system can detect an uncountable number of volatile molecules while retaining the ability to discriminate amongst very similar ones. We identified a unique mechanism employed by insect odorant receptors to discriminate amongst pheromones, chemical communication signals that orchestrate courtship and mating behavior. By coupling cryogenic electron microscopy (cryo-EM) and functional mutagenesis, we find that males of the silkmoth *Bombyx mori* distinguish between two quasi-identical compounds –bombykol, an alcohol, and bombykal, an aldehyde– by establishing a reversible covalent bond between the pheromone receptor and bombykal. Bombykol, instead, binds to the same receptor through hydrogen bonds, with significantly lower potency. The unique ability of aldehydes to establish a reversible covalent bond allows moths to unequivocally distinguish between compounds that differ only in the presence of a single hydrogen atom. Further, as many important odorants are aldehydes, this work illuminates a new binding mode available to the olfactory system to achieve high selectivity for these compounds.

## INTRODUCTION

An uncountable number of distinct odorant molecules reach the olfactory sensory organs of animals at any given time. The key role of the olfactory system is to translate this molecular complexity of the environment into interpretable neuronal signals, balancing the detection of a wide range of molecules against the accurate discrimination amongst very similar compounds [1–3]. To this goal, odorant receptors exhibit a wide range of ligand specificities: while most odorant receptors are promiscuous, responding to dozens of chemically diverse odorants, some receptors are uniquely activated by a single compound—often a cue that invariantly signals danger or suitability of a mating choice [3–6]. Promiscuous receptors enable combinatorial coding by allowing a relatively small number of receptors to encode an enormous variety of distinct smells. In contrast, narrowly tuned receptors selectively detect specific chemicals that carry critical information for survival, allowing for robust innate behavioral responses.

The sex communication system of insects is a canonical example of narrow tuning in the olfactory system. Insects emit volatile chemical signals that modulate key social interactions, including, courtship and mating. These sex communication chemical signals, termed ‘pheromones’, are often chemically related compounds with small variation across closely related species. Yet, insects have an exquisite ability to discriminate amongst these quasi-identical chemical compounds to make appropriate courtship decisions, literally, on the fly [5, 7, 8]. Insect pheromone receptors (PRs) are members of the insect odorant receptor (OR) family, with variable pheromone-tuning subunits that associate with a conserved co-receptor subunit called Orco [9–11]. PR/Orco complexes form ligand-gated heterotetrameric ion channels that are responsible for detecting pheromones and initiating a corresponding neuronal response [9, 10, 12–14]. Successful recognition of pheromones by PRs is thus crucial for these animals to detect their conspecific signal and maintain their reproductive isolation [15–19].

Sex pheromones in Lepidopterans –moths and butterflies– have received special attention. In some Lepidopteran species, adults exist nearly exclusively to disperse and reproduce, and therefore the majority of their olfactory system is tuned to the task of finding a suitable mating partner [20, 21]. Their reproductive success is facilitated by their ability to find mates under low population densities through long-range pheromonal communication, relying on exceptionally specific and sensitive PR/Orco complexes [8, 22].

**Figure 1.**
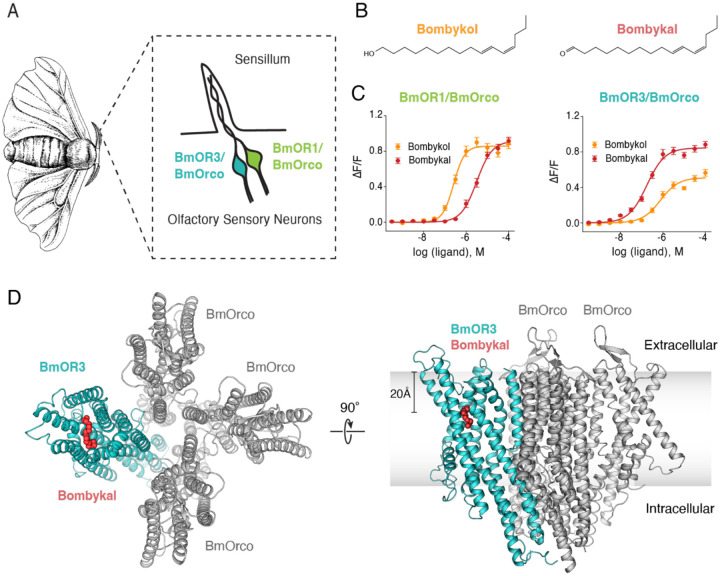
Bombyx mori pheromone recognition system. (**A**) Schematic of the pheromone sensing structure in a *Bombyx mori* male. The olfactory sensory neurons (OSNs) are housed in structures called sensilla in the moth antenna. Each pheromone-sensing sensillum houses two OSNs that express either the pheromone receptors BmOR1 or BmOR3. Both OSNs co-express the co-receptor BmOrco. (**B**) Chemical structures of bombykol (left) and bombykal (right). (**C**) Left, dose-response curves of BmOR1/Orco to bombykol (red) and bombykal (orange) and right, dose-response curves of BmOR3/Orco to bombykol (red) and bombykal (orange). (**D**) Cryo-EM structure of BmOR3/Orco shown from the top (left) and side (right). BmOR3 subunit is colored in teal, Orco subunits in grey, and bombykal in red. The grey gradient indicates the position of the cell membrane.

The exceptional chemical recognition capability of PRs is classically captured in the pheromone communication system of the domestic silk moth, *Bombyx mori* (Figure 1A) [9, 23–26]. Male moths of the species *B. mori* must detect bombykol, emitted by female moths of *B. mori*, and discriminate it from hundreds of similar chemical variants emitted by moths of other species. Most impressively, female moths of closely related species emit bombykal, a nearly-identical pheromone that differs only in its terminal functional group such that bombykol is an alcohol and bombykal is an aldehyde (Figure 1B) [27–30]. Therefore, their molecular formula differs solely in the presence of a hydrogen atom. Despite their chemical similarity, the two pheromones elicit entirely opposite behavioral responses in *B. mori*: bombykol causes conspecific male moths to exhibit aggressive attraction behavior, whereas bombykal suppresses mating behavior [18, 23, 24, 27, 31]. Thus, for these male moths, the ability to recognize their conspecific counterpart hinges on their PRs’ ability to discriminate between a hydroxyl and a carbonyl group.

Here, we discover a molecular strategy that enables exquisite chemical specificity in the olfactory system. We find that aldehyde volatile ligands can establish a reversible covalent bond with odorant receptors, a specific chemical interaction that enables unequivocal differentiation from volatile ligands containing other functional groups. *B. mori* moths combine this unique chemical interaction with strict geometric requirements that only accommodate a specific hydrocarbon chain to develop an effective chemical recognition strategy that enables appropriate behavioral responses in the pursuit of reproductive success.

## RESULTS

The first insect pheromone system characterized at the molecular level is that of *Bombyx mori*, a moth long known to navigate towards its mate guided solely by pheromone cues with impressive navigation accuracy [31]. Two PR/Orco complexes, BmOR1/BmOrco and BmOR3/BmOrco, have been linked to the behavioral responses to pheromones in *B. mori* (Figure 1A) [9, 17, 19, 23–25]. The conspecific pheromone, bombykol, is detected through BmOR1/Orco and leads to sexual attraction. The heterospecific pheromone, bombykal, is sensed through BmOR3/Orco, and leads to suppression of mating behavior. We first characterized the extent to which both PR/Orco complexes are selective towards their cognate ligands. We used a previously established calcium flux functional assay where we co-transfected HEK293 cells with either BmOR1 or BmOR3, along with the coreceptor, BmOrco (here on referred to as Orco), and the fluorescent calcium reporter, GCaMP6 (Figure 1C) [32, 33]. In this assay, ligand-activation of the heteromeric ion channel complexes by addition of either bombykol or bombykal leads to calcium influx, read out as GCaMP6 fluorescence changes that can be monitored in real-time and dose-dependent manner using a fluorescence plate reader. Our results confirmed that BmOR1/Orco is indeed more potently activated by bombykol over bombykal in EC_50_ (bombykol EC_50_ = 0.27 μM ± 0.05 μM, bombykal EC_50_ = 3.0 μM ± 0.5 μM) and that BmOR3/Orco shows higher activity towards bombykal over bombykol, both in EC_50_ and maximal activation (bombykol EC_50_ = 1.3 μM ± 0.8 μM, Max ΔF/F = 0.61 ± 0.04, bombykal EC_50_ = 0.16 μM ± 0.03 μM, Max ΔF/F = 0.98 ± 0.05) (Figure 1C and Table S1). But we also found both PR/Orco complexes to be somewhat promiscuous: both complexes are activated by their non-cognate pheromones (bombykal for BmOR1/Orco and bombykol for BmOR3/Orco) with a response that is ~10 fold smaller than their cognate pheromones. This result immediately suggests that a single receptor is unable to distinguish between bombykal and bombykal at all concentration ranges. Instead, two complexes with reciprocal specificities enable an unequivocal response to bombykal and bombykol across all concentrations, ensuring the behavioral accuracy needed for mate selection.

Bombykol and bombykal are long-chain hydrocarbons that differ only in the oxidation state of the terminal C-O group (Figure 1B). As such, the differentiation mechanism between the two ligands must emerge from specific recognition of their polar groups: an alcohol group in bombykol and an aldehyde in bombykal. Hydroxyl groups in alcohols can establish multiple hydrogen bonds, as both donors and acceptors. Carbonyl groups in aldehydes, unless hydrated, can act only as hydrogen bond acceptors. How, then, does BmOR3/Orco recognize the aldehyde, bombykal, with a lower EC_50_ than the equivalent alcohol, bombykol? An atomic view of pheromone binding by the receptor is needed to understand the mechanism of this pheromone preference.

### Structure of the bombykal receptor BmOR3/Orco

We purified the BmOR3/Orco heteromeric complex from Expi293F GnTI− cells (Figure S1) and determined its structure in the presence and absence of bombykal using single particle cryo-EM (Figure S2 and Table S2). As observed in recent cryo-EM studies of insect heteromeric ORs, both datasets contained two distinct conformations of BmOR3/Orco – open-pore and closed-pore – indicating the existence of an open/closed equilibrium, consistent with the high baseline GCaMP6 fluorescence of BmOR3/Orco in the absence of any odorants (Figures S2, S3). We did not make quantitative inferences on whether the distribution of cryo-EM particles in each state relates to the equilibrium between open and closed states, as the detergent environment does not mimic the native cell membrane.

We first focus on the open-pore, bombykal-bound, structure to gain insights on how interactions between bombykal and the receptor lead to channel opening. Three-dimensional reconstruction resulted in a density map with overall 2.61 Å resolution, which allowed us to build a model for the majority of the protein (Figures 1D, S4 and S5). Consistent with recent structural observations of OR/Orco complexes from other insect species [34, 35], we found that the heteromeric BmOR3/Orco complexes purified from heterologous systems have one OR subunit and three Orco subunits (Figure 1D). Whether other stoichiometries are possible in native tissues remains an exciting open question in the field.

All four subunits contribute to a central ion conduction pathway with a singular opening into the extracellular space. A wide vestibule then runs through the transmembrane region and opens into four intracellular exits (Figure S6). Most inter-subunit interactions localize to the intracellular ‘anchor’ domain, as is canonical in this family of proteins. However, compared with other members of the insect OR family, BmOR3 has an extended intracellular loop connecting helices S4 and S5 that packs tightly against the neighboring BmOrco subunit, providing an extra set of stabilizing inter-subunit interactions (Figure S7).

### A dual-recognition strategy enables modular recognition of the hydrocarbon tail and the polar function group

**Figure 2.**
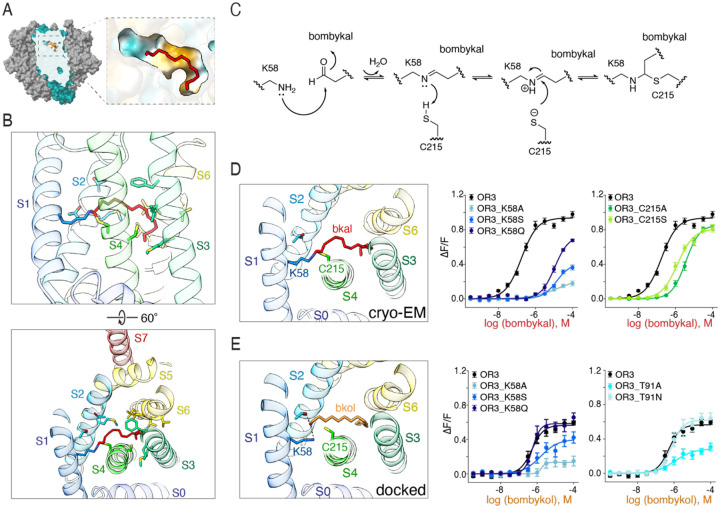
BmOR3/Orco recognizes bombykal through covalent bond formation. (**A**) Surface view of BmOR3/Orco, sliced through the OR3 subunit to show the binding pocket. Inset: detail of the binding pocket, colored by hydrophobicity. Acqua color depicts hydrophilic surfaces, yellow color marks hydrophobic surfaces. (**B**) Top: view of the binding pocket in the same orientation as in (**A**). Helices are colored in rainbow from the N-terminus (blue) to the C-terminus (red). Residues in contact with bombykal are shown and colored by helices. Bottom: top view of the binding pocket. (**C**) Schematic of the covalent linkage formation between the aldehyde functional group of bombykal and Lys58 and C215. (**D**) Left, zoomed in view of the binding pocket, showing bombykal and the polar residues that interact with the functional group of bombykal. Right, bombykal dose-response curves of BmOR3/Orco mutants targeting Lys58 and Cys215. (**E**) Left, binding pocket view of BmOR3/Orco with bombykol structure modeled through computational docking. Right, bombykol dose-response curves of BmOR3/Orco mutants targeting Lys58 and Cys215.

The canonical OR/Orco binding site observed in previous structural studies [33–35] is in a cavity ~20Å deep in the transmembrane region of the OR subunit, formed by convergence of the S2, S3, S4, and S6 transmembrane helices. In BmOR3/Orco, this region forms a cavity much larger than those of previously reported ORs, and reaches deeper towards the S1 helix (Figures 2A, B and S7). One end of this cavity, tucked against the S1 helix, is lined with charged and polar residues (Figure 2A). The bombykal density is strong and unambiguous and extends continuously from a lysine (Lys58) in S1 (Figure S8). The remainder of the cavity, towards the S6 helix, forms a hydrophobic tunnel with a terminal curl (Figure 2A). This cavity is occupied by an elongated density that matches the length and shape of bombykal. The aldehyde end of the molecule orients towards the polar pocket near the S1 helix, and the hydrocarbon end towards the hydrophobic cavity near the S6 and S3 helices (Figure 2B). Therefore, the elongated nature of the ligand enables a modular recognition strategy, where the main distinguishing features of the ligand –the polar group and the hydrophobic hydrocarbon chain– are detected at two spatially distinct regions of the binding pocket.

### A covalent linkage underlies recognition of the polar end of the pheromone by BmOR3/Orco

To understand the nature of the interaction between bombykal and BmOR3/Orco, we used molecular docking to place bombykal into the ligand-bound BmOR3/Orco structure (Figures S8A, B). All top binding poses of bombykal roughly fit into the observed ligand density but placed the aldehyde group sitting above Lys58, significantly deviating from the observed cryo-EM density and predicting no specific interactions between Lys58 and the bombykal aldehyde group. In contrast, docking the non-cognate pheromone bombykol into the same pocket yielded a similar pose, with the hydroxyl group predicted to form hydrogen bonding interactions with Lys58 and a neighboring Thr91 (Figure S8C). The molecular docking scores for bombykal and bombykol were nearly identical, suggesting that molecular docking is unable to explain the clear pheromone preference of BmOR3/Orco for bombykal over bombykol (Figure S8).

The chemical reactivity of aldehydes offers a solution to this apparent contradiction. Aldehydes are highly reactive as electrophiles compared to alcohols and can readily react with nucleophilic primary amine groups (such as those found in lysines) to form a Schiff base, a type of reversible covalent bond [36]. Formation of a Schiff base occurs when the primary amine of a lysine carries out a nucleophilic attack on the carbonyl carbon of the aldehyde, yielding an imine bond and expelling a water molecule (Figure 2C). A covalent bond between bombykal and Lys58 in BmOR3/Orco would give rise to a continuous density extending from Lys58 and the loss of the carbonyl oxygen during hydrolysis effectively shortens the chain length of the ligand. Indeed, we observed that covalent docking of bombykal into BmOR3/Orco yielded substantially improved docking scores and poses (Figure S8D): both the polar end of bombykal as well as the hydrophobic tail fit the observed density much better than without the covalent adduct (Figure S8B).

Given the crucial role of Lys58 in establishing a covalent bond specifically with bombykal but not with bombykol, we hypothesized that mutations of Lys58 would differentially disrupt BmOR3/Orco’s ability to respond to bombykol and bombykal. We replaced Lys58 with alanine, serine and glutamine, of which the last two can potentially establish hydrogen bonds but cannot form a Schiff base with the aldehyde group of bombykal. We found that all mutants abolished BmOR3/Orco’s activity towards bombykal (Figure 2D and Table S3). This suggests that despite introducing substitutions that can form hydrogen bonds, BmOR3/Orco cannot recognize bombykal without the primary amine of the lysine. In contrast, the same Lys58 mutations had a significantly different impact for bombykol-dependent activation of BmOR3/Orco: while the alanine mutation significantly attenuated activation, serine and glutamine progressively recovered the response of BmOR3/Orco to bombykol (Figure 2E and Table S3). These results show that BmOR3/Orco primarily interacts with bombykol through hydrogen bonds but requires the primary amine group of Lys58 to recognize its cognate pheromone, bombykal. The unique ability of aldehydes to form a Schiff base with the primary amine group of lysine thus underlies the bombykal preference by BmOR3/Orco.

A classic example of a Schiff base in a sensory process is the bond between retinal and rhodopsin that underlies light detection in retinal photoreceptor cells [37]. In the Schiff base formed between rhodopsin and retinal, the imine nitrogen is often protonated and stabilized by a neighboring, negatively charged glutamate [38]. In the absence of such charged residue, the Schiff base undergoes rapid hydrolysis and does not form a stable intermediate [39]. BmOR3/Orco does not have such charged residue to stabilize the Schiff base but instead, has a Cys215 residue positioned directly underneath the imine bond between the lysine and bombykal. The cryo-EM density displays clear continuity between Cys215 and the imine carbon, prompting us to consider two possible models for the role of Cys215 in Schiff bond stabilization. (i) The cysteine could stabilize the Schiff bond through a hydrogen bond between the sulfhydryl group of cysteine and the lone electron pair of the imine bond nitrogen. Alternatively, (ii) the cysteine, which is a nucleophile, could attack the imine bond carbon to form a thio-hemiaminal. To investigate these models, we explored the effects of a Cys215 mutation. Replacements of Cys215 with alanine or serine both showed a comparable decrease in bombykal-dependent activation of BmOR3/Orco (Figure 2D). As serine is a stronger hydrogen bond donor than cysteine, its inability to substitute for cysteine suggests that the more nucleophilic nature of the sulfhydryl group is required to stabilize the imine bond (Figure 2C). Consistent with this view, the geometry of a thio-hemiaminal is well captured by the observed cryo-EM density at this position (Figure S8E). Therefore, Cys215 likely serves a stabilization role by preventing premature hydrolysis of the Schiff base.

### A strict geometric requirement for activation of BmOR3/Orco confers selectivity towards the hydrophobic tail of bombykal

Lepidopteran pheromones are long chain hydrocarbons, often unsaturated, with diversified position and stereochemistry of the conjugated double bonds [22, 40]. The exquisite specificity of moth PR/Orco complexes for the correct hydrocarbon tail has been extensively documented at the functional level, prompting us to evaluate its structural correlate [9, 22, 26, 30, 41].

**Figure 3.**
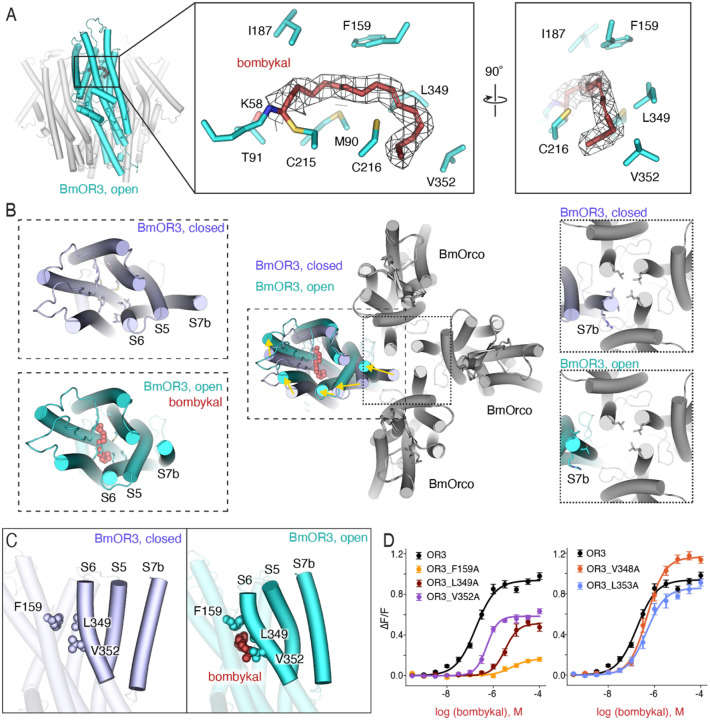
Hydrophobic residues mediate recognition of the hydrophobic tail of bombykal and receptor activation. (**A**) Sideview of the bombykal bound BmOR3/Orco complex. Insets, close-up views of the ligand binding pocket showing the residues interacting with bombykal. (**B**) Middle, overlay of the unbound and bombykal-bound BmOR3/Orco complex in a top view, displaying the structural rearrangements that occur during pore opening. Left inset, focused comparison of structural differences in BmOR3 between the closed (top) and open state (bottom). Right inset, close-up of the pore showing displacement of the S7b helix in BmOR3. (**C**) A close-up lateral view of S6, S5, S7b helices in BmOR3 in its closed (left) and open (right) states. Also shown are important residues in S6 (Leu349 and V352) and S3 (F159) that interact with the hydrophobic tail of bombykal and mediate activation. (**D**) Bombykal dose-response curves of BmOR3/Orco mutants targeting residues involved in hydrophobic tail recognition and receptor activation (left), or adjacent residues on S6 which do not interact with bombykal (right).

In the bombykal-bound structure of BmOR3/Orco, the hydrophobic tail of bombykal is tucked neatly into a hydrophobic tunnel oriented towards the S6 helix. Bombykal density adopts a planar hook shape dictated by the position and stereochemistry of the conjugated dienes in the hydrocarbon chain, a geometry that is perfectly complemented by the arrangement of hydrophobic and aromatic residues in the binding pocket (Figure 3A).

The conjugated dienes of bombykal are positioned towards the S6 helix in the binding pocket, a region that has been previously implicated in dictating activation of the insect odorant receptor family [33–35]. The orientation of bombykal in the binding pocket suggests that correct placement of the hydrocarbon tail determines engagement of residues in S6 for channel activation. We thus elucidated the cryo-EM structure of BmOR3/Orco in the absence of ligand (Figures S2, S4, S5), to identify conformational changes that link bombykal binding and pore opening, and shed light on the role of hydrocarbon tail recognition.

We obtained a reconstruction of the closed pore state at overall 2.74Å resolution. In the closed-pore, bombykal-free structure, the ligand binding pocket was partially occupied by a small density, substantially smaller than bombykal, and located towards the polar end of the binding pocket, likely representing a small organic molecule or ions that interact with the polar end of the pocket but, lacking the long, hydrophobic tail of the cognate ligand, fail to elicit channel activation (Figure S5).

Consistent with previous structural studies on heteromeric insect odorant receptors [34, 35], pore opening is driven by conformational changes in the BmOR3 subunit alone, where the pore helix S7b is displaced diagonally away from the central pore axis (Figure 3B). This displacement of the helix S7b is driven by the interactions that the aliphatic tail of bombykal forms with the helix S6 of BmOR3. While helix S7b does not directly line the binding pocket, it is coupled with the helix S5, and helix S5 with helix S6, through a network of interactions that translate any displacement of the binding pocket helix S6 into a displacement of pore helix S7b (Figure 3C). When BmOR3/Orco is bound to bombykal, two residues in helix S6 –Leu349 and Val352– form hydrophobic contacts with bombykal. Leu349 stacks against the conjugated diene of bombykal and Val352 contacts the end of the hydrocarbon tail. Alanine mutations of both residues significantly attenuate BmOR3/Orco response to bombykal, indicating that these interactions are crucial for stabilizing the open conformation (Figure 3D, left and Table S3). In contrast, mutations of neighboring helix S6 residues Val348 and Leu353, which do not form hydrophobic contacts with bombykal, do not affect BmOR3/Orco activity (Figure 3D, right and Table S3). This mechanism of activation explains why bombykal elicits a higher maximal activation of BmOR3/Orco, compared to bombykol (Figure 1C and Table S1): binding of BmOR3/Orco to bombykal through a Schiff base results in a short bond between Lys58 and the imine carbonyl, effectively bringing the bombykal molecule closer to the Lys58 and positioning the conjugated diene to perfectly engage Leu 349. No amount of bombykol can elicit full activation of BmOR3/Orco, likely because binding through hydrogen bonds results in a longer binding geometry of the polar group, with a poor fit of the hydrophobic chain and suboptimal engagement of Leu349.

Interaction of the hydrophobic tail of bombykal with these key residues in S6 depends on the proper fit of the ligand, defined by the position and stereochemistry of its conjugated diene (Figure 3A). When bombykal binds with its aldehyde group anchored at Lys58 in S1, the conjugated diene of bombykal creates a kink that orients the hydrocarbon chain such that it fits properly in this hydrophobic pocket of BmOR3 and forms crucial contacts with residues Leu349 and Val352 in S6 helix. The S6-S5-S7b helix bundle displaces towards the binding pocket, and residues in helices S3, S4, and S6 form a constricted hydrophobic pocket that encapsulates the aliphatic tail. Phe159 in helix S3 forms a cap and residues Met90, Cys216 and Leu349 form the side walls of the pocket. Mutation of Phe159 to alanine sharply ablates receptor function (Figure 3D and Table S3).

This mechanism of receptor activation by engagement of the hydrophobic end of the pocket explains the rigid geometric constraints known to be required for *B. mori* pheromone recognition [9, 26]. By imposing a dual requirement for both its unique covalent interaction with the aldehyde and the geometric constraints for the stereospecific hydrophobic tail, BmOR3/Orco achieves selectivity towards bombykal.

### BmOR1/Orco and BmOR3/Orco have conserved pheromone binding pockets but distinct recognition strategies

**Figure 4.**
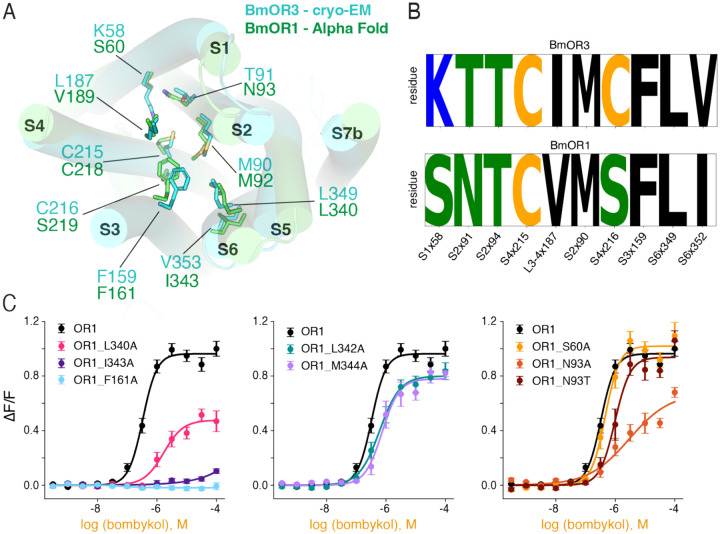
BmOR1 and BmOR3 share a common binding mode but diverging recognition strategies. (**A**) Structural alignment of the cryo-EM structure of BmOR3 (teal) with an AlphaFold predicted structure of BmOR1 (green). (**B**) Sequence logo for BmOR1 and BmOR3 at select residues outlining the binding pocket, indicating the occupancy at each position in BmOR3 and BmOR1. (**C**) Bombykol dose-response curves of BmOR1/Orco mutants targeting residues outlining its putative binding pocket, based on comparison with the cryo-EM structure of BmOR3/Orco.

We next leveraged our study of BmOR3/Orco as a structural framework to evaluate how BmOR1/Orco might recognize its cognate ligand, bombykol. Because the two PRs share an extremely conserved 3D structure, superposition of the BmOR3 subunit from our bombykal bound BmOR3/Orco structure with an Alphafold model of BmOR1 subunit (Figure 4A, RMSD=1.6 Å) allowed accurate comparison between the residues lining their binding pockets.

The binding pockets of BmOR1 and BmOR3 show conservation in the residues that recognize the hydrophobic tail of bombykol and bombykal (Figure 4A, B). We introduced several mutations in BmOR1/Orco predicted to disrupt these hydrophobic interactions and evaluated their impact in BmOR1/Orco response towards its cognate pheromone bombykol (Figure 4C and Table S3). Alanine mutations of Phe161 (Phe159 in BmOR3), Leu340 (Leu349 in BmOR3), and Ile343 (Val352 in BmOR3) all significantly attenuated receptor activity, mirroring the role of the corresponding residues in BmOR3/Orco (Figure 4C, left). Moreover, mutation of neighboring residues Leu342 and Met344, which are not predicted to interact with bombykol, had minimal impact on receptor activity (Figure 4C, middle). The conserved functional roles of these key residues in ligand-dependent receptor activity indicate that BmOR1 and BmOR3 share a common mechanism of hydrophobic tail recognition and channel activation, and indicate that Alphafold predictive models of BmOR1 can be combined with experimental data obtained in BmOR3 to confidently evaluate its ligand binding.

BmOR1 and BmOR3 diverge in two residue positions in the polar end of the pocket, which in BmOR3 serves as the site of recognition of the polar functional group of the ligand (Figure 4A, B). Residues Lys58 and Thr91 in BmOR3 correspond to Ser60 and Asn93, respectively, in BmOR1. Mutation of Asn93 to alanine significantly lowered the activity of BmOR1/Orco in response to bombykol, whereas mutation of Ser60 to alanine did not affect receptor activity, suggesting that BmOR1 primarily recognizes bombykol through Asn93 (Figure 4C, right). Furthermore, mutation of Asn93 to threonine, which can form hydrogen bonds, fully recovered receptor activity, indicating that Asn93 likely recognizes the alcohol group of bombykol through hydrogen bonding interactions (Figure 4C, right).

Such divergence provides key insights to how BmOR1 and BmOR3 have evolved to tune their selectivity towards their cognate ligands bombykol and bombykal, respectively. BmOR1 recognizes bombykol through Asn93 primarily through hydrogen bonding interactions. In turn, BmOR3 recognizes bombykal by forming a covalent linkage between the aldehyde and Lys58, which is a unique interaction that only aldehydes can form. Amino acid diversification at key positions in the polar end of the binding pocket therefore enables surgical precision in the recognition of two nearly-identical molecules, allowing *B. mori* PR/Orco complexes to unequivocally tune their preference for their cognate pheromone ligands.

## DISCUSSION

In the olfactory system, the narrow tuning properties of select odorant receptors are central to enable unequivocal detection of ecologically important cues. In this work, we elucidate how a pair of *B. mori* PRs: BmOR1 and BmOR3 each tune their selectivity to preferentially recognize their cognate pheromones. A two-factor recognition strategy, where the specific terminal functional group and the stereospecific hydrophobic tail of the cognate pheromone are modularly recognized by distinct regions of the binding pocket, enables the high selectivity required to discriminate amongst highly similar pheromones. Further, a reversible covalent bond –between a lysine in the BmOR3 binding pocket and the aldehyde ligand– enables discrimination amongst two quasi-identical compounds, a new chemical strategy for odorant discrimination. Because many odorants are aldehydes and many orphan odorant receptors contain lysines in the predicted binding pockets, we suspect reversible covalent bond formation might be a widespread strategy for odorant discrimination of this chemical class.

This dual recognition strategy of narrowly tuned Lepidopteran PRs –with a polar functional group recognized by one end of the pocket, and the length and shape of the hydrophobic tail requiring a tight geometric fit– represents a distinct mechanism, divergent from that observed for broadly tuned, promiscuous ORs. In the broadly tuned ORs structurally characterized thus far, odorants bind the receptor through flexible, hydrophobic interactions that allow diverse odorant ligands to occupy a variety of poses [33, 34, 42]. PRs, instead, impose a strict binding mode, more closely resembling the classic lock-and-key binding mechanism. Both the polar end of the pheromone, fixed towards the S1 helix, and the hydrophobic tail, facing the S6 helix, require precise complementarity in chemistry and geometry in order to activate the receptor.

**Figure 5.**
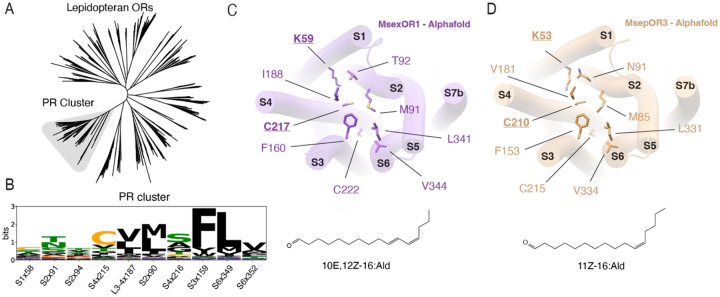
A conserved binding locus across Lepidopteran PRs. (**A**) Unrooted phylogenetic tree of Lepidopteran ORs. Grey highlight corresponds to branch containing the PR subfamily. (**B**) Sequence logo for 249 Lepidopteran PRs, all in the PR cluster. The size of each letter is proportional to its representation at the indicated position. Black, hydrophobic residues. Green, polar residues. Red, acidic residues. Blue, basic residues. Cysteine, orange. (**C**)(**D**) Alphafold structure displaying the binding pocket residues of (**C**) MsexOR1 and (**D**) MsepOR3. Corresponding cognate pheromone ligands are shown below.

Lepidopteran pheromones share a common chemical structure consisting of a long chain hydrocarbon (C_10_-C_18_) capped with a polar functional group of either an alcohol, aldehyde or acetate [14, 40]. Analysis of the conservation in binding pockets of PRs across the Lepidopteran clade suggests that our findings from the *B. mori* pheromone system likely represent a general recognition strategy across the clade (Figure 5A, B; see SI Materials and methods section for details of the structural alignment). In our comparison of 249 AlphaFold predicted structures of all Lepidopteran PRs, residues corresponding to positions Met90, Phe159, Ile187, Leu349, and Val352 –which all surround the hydrophobic tail in BmOR3–, were invariantly hydrophobic across all PRs (Fig 5B). Among these residues, Phe159 and Leu349 were particularly well conserved, suggesting a conserved role in recognition of the hydrophobic tail and receptor activation for all PRs. In contrast, residues corresponding to positions Lys58, Thr91, and Thr94, which line the pocket towards helix S1 and mediate recognition of the functional group in BmOR3 and BmOR1, were polar and divergent (Fig 5B). PRs likely employ varying combinations of residues in this region to recognize the various polar functional groups of their cognate pheromones.

Pheromones and their receptors play a key role in the evolutionary path towards speciation [15, 16, 22]. The chemical recognition strategy described in this work offers a simple structural path for how Lepidopteran PRs might have re-tuned their selectivity towards diverse pheromone ligands. Point mutations in key amino acids in the binding pocket can easily re-tune the PR selectivity towards individual features of the pheromones to enable the development of reproductive barriers between closely related Lepidopteran species. This approach lowers the evolutionary barrier to re-tune the selectivity of a PR for small chemical variation in pheromones, while maintaining the overall recognition towards the shared features of the pheromones within the clade.

Lepidopteran species are invasive agricultural pests that cost nearly $10 bn in annual losses globally [43, 44]. Our discovery of a covalent mechanism of pheromone recognition hints at an exciting opportunity to develop effective attractants and repellents against these harmful pests. Covalent inhibitors are widely used in medicinal chemistry to generate ligands with exceptional selectivity and activity towards targeted proteins [45–48]. These drugs use electrophilic groups, often termed as “warheads”, that form reversible or irreversible covalent bonds with specific amino acid residues to potently target receptors. Among targeted residues, cysteines and lysines are the most common sites of covalent attachment, which are widely found in the binding pockets of many known PRs, and can be leveraged to target many Lepidopteran species that cause particularly harmful agricultural damage. Based on our structural alignment of PRs, position 58 (Lys58 in BmOR3), the crucial residue for aldehyde recognition by BmOR3, is occupied by lysines in two other PRs: *Manduca sexta* OR1 (Figure 5C) and *Mythimna separata* OR3 (Figure 5D). For both PRs, the cognate pheromone ligands are aldehydes –bombykal for MsexOR1 [29] and Z11–16:Ald for MsepOR3 [49]– suggesting the possibility that they, too, rely on a covalent adduct to recognize their aldehyde pheromones (Figure 5C,D). Of these two species, *M. sep* moths are major agricultural pests of several cereal crops that is found across Asia and Australasia, and their PR is a prime candidate for developing a covalent inhibitor to curb their population [50, 51].

In our current understanding of the olfactory system, the transient binding of an odorant molecule to an odorant receptor enables the initiation of a short-lived neuronal signaling event, whose macroscopic duration can be modulated by varying the concentration of odorant molecules in the odor plume. The establishment of a covalent bond between a pheromone receptor and its cognate ligand –even if said covalent bond is low-energy and therefore relatively reversible– often result in much slower unbinding kinetics [52], suggesting that the resulting neuronal signaling mechanism could exhibit a different kinetic signature. This observation opens an exciting prospect where the pharmacology of diverse ligand-receptor interactions could substantially modulate odor-driven navigation. Future work connecting the kinetics of pheromone receptor activation by covalently bound aldehyde ligands to the neuronal spiking rates and the animal’s navigation mechanisms, will shed light on the behavioral consequences of diverse types of ligand-receptor interactions.

## Supplementary Material

Supplement 1

## Data Availability

The cryo-EM density maps and the atomic models of the complexes have been deposited in the Electron Microscopy Data Bank and Protein Data Bank with the following accession numbers EMD-XXXXX and PDB: XXXX (BmOR3/Orco, apo), EMD-XXXXX and PDB:XXXX (BmOR3/Orco, bombykal bound. All other data needed to evaluate the conclusions in the paper are present in the paper and/or the supplementary materials.
