# Supplementary material for: A covalent recognition strategy enables conspecific mate identification
Source: bioRxiv. 2025 Jun 3:2025.05.30.657074. Preprint. [Version 1] doi: 10.1101/2025.05.30.657074 (PMC12157555; doi:10.1101/2025.05.30.657074)
Supplement: Supplement 1 [file media-1.pdf]

SUPPLEMENTAL INFORMATION

Sukjin S. Jang[1], Sanjana Mandala[1,4], Hanjie Jiang[1,2], Xiao Zhang[3], Phillip A. Cole[1,2] and Josefina del Mármol[1,4]

[1]Department of Biological Chemistry and Molecular Pharmacology, Harvard Medical School, Boston, MA, US

[2] Division of Genetics, Department of Medicine, Brigham and Women's Hospital, Boston, MA, US

[3]Department of Chemistry, Massachusetts Institute of Technology, Cambridge, MA, US

[4]Howard Hughes Medical Institute, Boston, MA, US

[*]Correspondence josefina_delmarmol@hms.harvard.edu

Materials and methods

Synthesis of Bombykal ((10E,12Z)-hexadeca-10,12-dien-1-al)

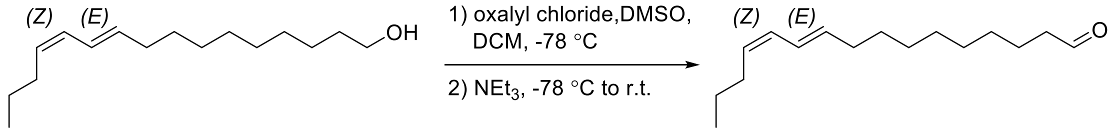

Bombykal was synthesized from commercially available bombykol ((10*E*,12*Z*)-hexadeca-10,12-dien-1-ol, MedChemExpress) following a modified literature procedure [1, 2]. To a solution of oxalyl chloride (8.6 µL, 0.1 mmol, 1.2 eq) in dichloromethane (1.2 mL) was added DMSO (12 µL, 0.168 mmol, 2.0 eq) at –78 °C to form a white suspension. After 10 minutes, a solution of bombykol (20 mg, 0.084 mmol, 1 eq) in dichloromethane (0.22 mL) was added dropwise to the reaction mixture at –78 °C and kept stirring for 30 min which resulted in a colorless solution. Triethylamine (70 µL, 0.5 mmol, 6.0 eq) was then added, and the reaction mixture was allowed to warm to room temperature. After 30 minutes, H_2O (10 mL) was added, and the aqueous phase was extracted with dichloromethane (10 mL, repeated three times). The combined organic phase was dried over sodium sulfate, filtered, and concentrated. The residue was purified by flash column chromatography on silica gel (using 1% to 2% ethyl acetate/hexane as eluent) to afford bombykal as colorless oil (99%, 20 mg). ^{1}H NMR (500 MHz, $CDCl_3$) δ 9.76 (t, J = 1.9 Hz, 1H), 6.34 – 6.25 (m, 1H), 5.95 (dd, J = 10.9, 10.7 Hz, 1H), 5.65 (dt, J = 14.6, 7.0 Hz, 1H), 5.30 (dt, J = 11.0, 7.6 Hz, 1H), 2.41 (td, J = 7.4, 1.9 Hz, 2H), 2.17 – 2.11 (m, 2H), 2.11 – 2.05 (m, 2H), 1.68 – 1.56 (m, 2H), 1.43 – 1.19 (m, 12H), 0.92 (t, J = 7.4 Hz, 3H). ^{13}C NMR (126 MHz, $CDCl_3$) δ 203.07, 134.71, 130.04, 128.90, 125.84, 44.05, 32.98, 29.89, 29.50, 29.44, 29.41, 29.29, 29.27, 23.04, 22.21, 13.93. The NMR spectra agree with the reported data [3].

Expression and purification of BmOR3/BmOrco complex

The coding sequence of BmOrco was a gift from Leslie Vosshall (Addgene plasmid #73926; http://n2t.net/addgene:73926 ; RRID:Addgene_73926). The coding sequence of BmOR3 was codon optimized and synthesized from Twist Bioscience. Gene fragments of full-length BmOR3 was cloned into a pEG BacMam vector containing an N-terminal superfolder GFP and an HRV 3C protease site. The gene fragment of full length BmOrco was cloned into a pEG BacMam vector containing an N-terminal mCherry and an HRV 3C protease site. Plasmids were transfected (1:2 ratio of BmOR3 to BmOrco, 750 µg per L) into Expi293F GnTI− cells grown in Expi293 medium with FectoPro (800 µL per L of culture) and kept at 37 °C with 8% carbon dioxide. After 18-24 h ,3 mM valproic acid and 0.5 % glucose were added, and the temperature was dropped from 37 °C to 30 °C for an additional 48-72 hours. Cells were then pelleted by centrifugation, flash frozen, and stored at −80 °C until the day of purification.

For purification, cell pellets were resuspended in 100 mL of ice cold solubilization buffer per liter of cell culture. The solubilization buffer was composed of 20 mM HEPES/NaOH

(pH 7.5), 150 mM NaCl, 0.5% (w/v) Lauryl Maltose Neopentyl Glycol (LMNG; Anatrace), 0.1% (w/v) cholesterol hemisuccinate (CHS; Sigma-Aldrich), 1 μg/mL leupeptin, 1mM benzamidine, 1 μg/mL aprotinin, and 1 μg/mL pepstatin A, and 1 mM phenylmethylsulfonyl fluoride (PMSF). The cells were kept in the cold room while rotating for 2 hours to extract the membrane protein complexes from the cell membranes to the micelle environment. The mixture was then clarified by centrifugation at 175,000 x g for 40 minutes, and the supernatant was added to 1 mL of anti-GFP nanobody-coupled Sepharose resin (bead volume) [4] per liter of cell culture. After washes with the Equilibration buffer (20 mM HEPES pH 7.5, 150 mM NaCl, 0.002% LMNG, 0.0004% CHS), the OR/Orco complex was eluted by mixing 50 μg of 3C protease (Sigma-Aldrich) with every 1 mL of resin and gentle rotation at 4 °C for an hour. The sample was then concentrated and injected into a Superose 6 Increase column (Cytiva) pre-equilibrated with the Equilibration buffer. Peak fractions containing BmOR3/Orco complex were pooled and concentrated to A280 = 2.74. The proteins were flash-frozen and stored at −80 °C until use.

CryoEM sample preparation and data acquisition

To prepare the bombykal-bound sample, bombykal was added to the OR3/Orco sample to a final concentration of 20 μM and incubated for an hour at room temperature. The apo sample omitted this step and was directly used for freezing grids. Cryo-EM grids were frozen using a Vitrobot Mark IV (FEI) using the following procedure: 4 μL of the sample was applied to a glow-discharged UltraAufoil R1.2/1.3, 300 mesh gold grid, blotted for 5 s in 100% humidity at 4 °C and Blot force: 1, and plunge frozen in liquid ethane cooled by liquid nitrogen.

Cryo-EM data were recorded on a 300-kV Titan Krios G3i microscope (ThermoFisher), equipped with a Gatan BioQuantum GIF/ K3 direct electron detection camera at the Janelia Research Campus Cryo-EM Facility. SerialEM was used for automated data collection. The defocus range is -1.0 to -2.0 μm. Movies were collected at a magnification of 81,000x in superresolution mode with a physical pixel size of 1.061 Å/pixel. All data was collected with a 30° tilted stage. 50 frames were collected with a total dose of 50 electrons per Å^2. Further details of the data collection parameters are listed in Supplementary Table 1.

CryoEM data processing

BmOR3/Orco with Bombykal: A total of 6,320 movies were collected for the OR3 sample with bombykal. The movies were aligned using motion corrected algorithms in CryoSPARC [5], and Blob-based autopicking in CryoSPARC [5] was used to select initial particles with 2-fold binning, resulting in 4,498,756 particles. From the initial 2D classification, a total of 2,934,701 particles were selected to reconstruct the initial map of the BmOR3/Orco complex by running an Ab-initio job followed by a homogeneous

refinement job. Using this reconstruction as a starting point, a seed-based method previously described in [6, 7], which involves iterative rounds of Ab-initio reconstruction and heterogeneous refinement were ran to remove false-positive particles and rescue ‘good’ particles that were excluded during the initial 2D classification. This resulted in a stack of 3,283,431 particles. Following non-uniform refinement, the particles were then classified through 3D classification in CryoSPARC [5] using a focused mask on the OR subunit. The 3D classification classified the particles to subsets representing two open-pore classes and two closed-pore class. Further processing of one of the open-pore classes (1,070,550 particles) through non-uniform refinement and local refinement generated a map with an overall resolution of 2.61 Å with clear bombykal density.

BmOR3/Orco without ligand: A total of 5,389 movies were collected for the OR3 sample in the absence of ligand. The movies were aligned using motion corrected algorithms in CryoSPARC [5], and Blob-based autopicking in CryoSPARC [5] was used to select initial particles with 2-fold binning, resulting in 4,652,403 particles. From the initial 2D classification, a total of 2,543,759 particles were selected to reconstruct the initial map of the BmOR3/Orco complex by running an Ab-initio job followed by a homogeneous refinement job. Using this reconstruction as a starting point, a seed-based method previously described in [6, 7], which involves iterative rounds of Ab-initio reconstruction and heterogeneous refinement were ran to remove false-positive particles and rescue ‘good’ particles that were excluded during the initial 2D classification. This resulted in a stack of 2,445,450 particles. Following non-uniform refinement, the particles were then classified through 3D classification in CryoSPARC [5] using a focused mask on the OR subunit. The 3D classification classified the particles to subsets representing two open-pore classes and two closed-pore class. Further processing of one of the closed-pore classes (327,260) that includes an additional round 3D classification, non-uniform refinement and local refinement generated a map with an overall resolution of 2.74 Å. Using the two unfiltered half-maps resulting from this local refinement job, a density modified map was generated in Resolved-cryoEM in Phenix [8] for model building.

Model Building

The Alpha Fold models [9, 10] of BmOrco (AF-Q7YT34-F1-v4) and BmOR3 (AF-Q5FBE0-F1-v4) were used as a starting point for manual model building in Coot [11]. Individual amino acid residues were assigned based on the quality of the side-chain densities in the primary maps.

The structure of bombykal bound, open conformation of BmOR3/Orco was built into the sharpened map. For the BmOR3 subunit, N-terminal residues 1-4 and the loop residues connecting S4-S5, 256-275 were omitted due to poor density in the map. For the Orco subunits, N-terminal residues 1-6 and intracellular S4-S5 loop residues 250-318 were omitted due to poor density in the map. Atomic coordinates were refined against the

sharpened map using real space refinement implemented in PHENIX [12] for 5 macrocycles with secondary structure restraints applied and without symmetry enforced. The bombykal bound model was refined including the ligand, with restraints obtained using eLBOW implemented in Phenix [12]. A separate edits parameter file was supplemented during the refinement to manually define the covalent linkage between bombykal and Lys58 and Cys215.

The structure of the apo, closed conformation of BmOR3/Orco was built into the density-modified map. For the BmOR3 subunit, N-terminal residues 1-4 and the loop residues connecting S4-S5, 256-279, and the loop residues connecting S3-S6, 339-343 were omitted due to poor density in the map. For the Orco subunits, N-terminal residues 1-6, extracellular S3-S4 loop residues 169-172, and intracellular S4-S5 loop residues 250-319 were omitted due to poor density in the map. Atomic coordinates were refined against the density-modified map using real space refinement implemented in PHENIX [12] for 5 macrocycles with secondary structure restraints applied and without symmetry enforced.

All structural biology software were compiled by SBGrid [13].

Molecular Docking

Non-covalent docking of bombykol and bombykal to BmOR3 was carried out using Glide implemented in Maestro (Schrödinger) [14]. The bombykal-bound structure of BmOR3/Orco was imported and were prepared for docking in Maestro. The bombykol and bombykal ligand structures were imported into Maestro using their SMILES notation and prepared using Epik Classic to generate their possible tautomeric and ionization states, while being optimized at pH 7.0 ± 2. The binding pocket was determined using SiteMap and a cubical grid search was built centered around the region of the highest-scoring site and was set to dock ligands of similar length to the Sitemap density. All ligands of interest were docked within the grid, and the top poses are presented in (Fig S7).

For covalent docking, the top scoring docking pose resulting from the non-covalent docking of bombykal was used as a starting point. In CovDock [15], bombykal was used to define the initial grid density and Lys58 was designated as the reactive residue to undergo Imine condensation with the aldehyde group of bombykal to form a Schiff Base. The output poses and their corresponding docking scores are presented in (Fig S7).

Pore analysis

Pore diameter along the central axis and side exits were calculated using HOLE [16]. For each structure, separate calculations were conducted for the central pore and the lateral conduits by varying the starting position and the vector defining the orientation of the pore finding.

GCaMP6 fluorescence calcium flux assay

The assay was performed similarly to method descriptions in previous studies [7, 17, 18]. All DNA constructs used in this assay were cloned into a modified pME18 vector that contains a SV40 promotor.

Each transfection condition (per well) contained a total of 300 ng of plasmids in 1:1:1 ratio of the GCaMP6, Orco, and the corresponding OR plasmid that was diluted in 4.8 uL of OptiMEM (Gibco) along with 0.1 uL of P3000. The mixture was incubated for 5 minutes and then further incubated for 20 minutes after being mixed with Lipofectamine 3000 (Invitrogen) diluted in OptiMEM. This mixture was scaled accordingly to batch transfect 50-100 wells per construct per plate.

HEK293T cells were maintained at 37°C with 5% carbon dioxide and grown in high glucose DMEM enhanced with 10% FBS and 1% GlutaMAX (Gibco). Cells were detached with TrypLE Express Enzyme and resuspended in FluoroBrite DMEM (Gibco) with 10% FBS and 1% GlutaMAX (Gibco) to a concentration of 5×10^5 cells/ml. Cells were combined with each transfection condition and added to 2x16 wells of a 384-well plate (Grenier CELLSTAR). Cells were kept at 37°C for 18-20 hours before being used for fluorescence plate reading.

Odorant plates were prepared using D300e (Hewlett Packard), a digital, non-contact dispenser. Briefly, stock solutions of bombykol and bombykal were prepared in DMSO and plated onto 384-plates (Greiner, Catalog #784201) in a titrating volume to obtain a concentration series ranging between 316 nM and 100 mM with a final volume of 0.2 μL. The odorant plates were kept at −20 °C until usage.

On the day of the GCaMP6 assay, Bravo (Agilent), a versatile liquid handler was used to prepare both the odorant plate and the assay plate (transfected cell plate) prior to the fluorescence readout using the FDSS/μcell kinetic plate Imager (Hamamatsu, C13299). For the odorant plate, the DMSO dissolved stock solutions were diluted in 19.8 μL of Reading buffer (20 mM HEPES/NaOH (pH 7.4), 1× HBSS (Gibco), 3 mM Na_2CO_3, 1 mM $MgSO_4$, and 5 mM $CaCl_2$) to give a final concentration ranging from 3.16 nM to 1 mM with 1% DMSO. For the cell plate, the FluoroBrite DMEM (Gibco) was exchanged out with the Reading buffer.

The kinetics of the fluorescence change upon odorant addition were measured using the FDSS/μcell kinetic plate Imager (Hamamatsu, C13299). Excitation was at 480 nm and emission was recorded at 540 nm. The exposure time was set to 0.5 s and the LED power was set to 400 mA. After 20 s of baseline recording, 4 μL of odorant solution was added to each well of the assay plate containing 36 μL of the Reading buffer followed by brief mixing, and the fluorescence of each well was continuously recorded for additional 5-10 minutes. All recordings were carried out at room temperature.

Each plate contained a negative control of GCaMP6 transfected alone or GCaMP6+Orco and exposed to the pheromone ligands. Additionally, each plate included either BmOR1/Orco or BmOR3/Orco with its corresponding pheromone ligand as a positive control to account for the variability in transfection efficiency and cell count. Each concentration of ligand was applied to two technical replicates, which were averaged and considered a single biological replicate. The baseline fluorescence, F_0, was determined using the first 10 s of GCaMP6 recording, prior to the addition of ligands. ΔF was calculated by taking the average of F values in the last 10 s of the fluorescence time trajectory and subtracting by the F_0. $\Delta F/F_0$ was then calculated for each well and further corrected by subtracting the $\Delta F/F_0$ obtained from three control wells of the same transfection condition but without any ligand addition. These normalized $\Delta F/F_0$ were averaged across the biological replicates to obtain the representative $\Delta F/F_0$ for each concentration point of the specific construct. Finally, custom written python scripts were used to fit the dose-response curves to the two-parameter Hill equation, from which EC_{50} and hill coefficient values were extracted. Max $\Delta F/F$ was defined as the $\Delta F/F$ at the highest concentration of the respective ligand in a given dose response curve.

For all mutant BmOR3/Orco complexes, expression levels were inferred from the baseline fluorescence before addition of the ligand as this metric is a direct indicator of channel activity in the absence of ligand. Baseline fluorescence for all BmOR3/Orco complexes was significantly above the GCaMP6 only baseline fluorescence and comparable to the wildtype fluorescence, indicating that they express and form heterotetrameric complexes (Fig S3). The differences in dose-response activation curve are likely to represent differences in receptor binding and channel activation properties, rather than deficiencies in the folding or trafficking pathways.

Sequence conservation across Lepidopteran ORs

From the uniprot database, FASTA files for Lepidopteran ORs were downloaded with the following search parameters: Taxonomy 7088, OR lengths between 350-500, odorant receptor, olfactory receptor, NOT coreceptor. This list was then combined with a list of uniprot accession codes with candidate PRs that were identified in [19]. Duplicate OR fasta files were removed using CD-HIT [20] by removing overlapping sequences with sequence similarity over 90%. The filtered set of uniprot IDs were then searched in the alphafold database to obtain 1366 unique Lepidopteran OR PDB files.

We then used FoldMason [21] to generate a multiple structural alignment (MSTA) of these receptors. Phylogenetic tree was then built using IQ-Tree2 with 1000 ultrafast bootstrap replicates [22]. The tree was visualized with iTOL(Interactive Tree of Life) [23]. A single cluster, of 249 ORs which contained 180 of the known PRs was annotated as the PR cluster. These receptors were then subsequently re-aligned in FOLDMASON [21] to output the MSTA file used for observing sequence conservation pattern across the binding pocket residues in Lepidopteran PRs. The average Local Distance Difference Test (LDDT) score, a metric of the quality of the structural alignment, for all core domains of

these PRs was 0.849, meaning that they share a highly conserved fold and conservation across residues within these domains can be assessed confidently.

Figure S1

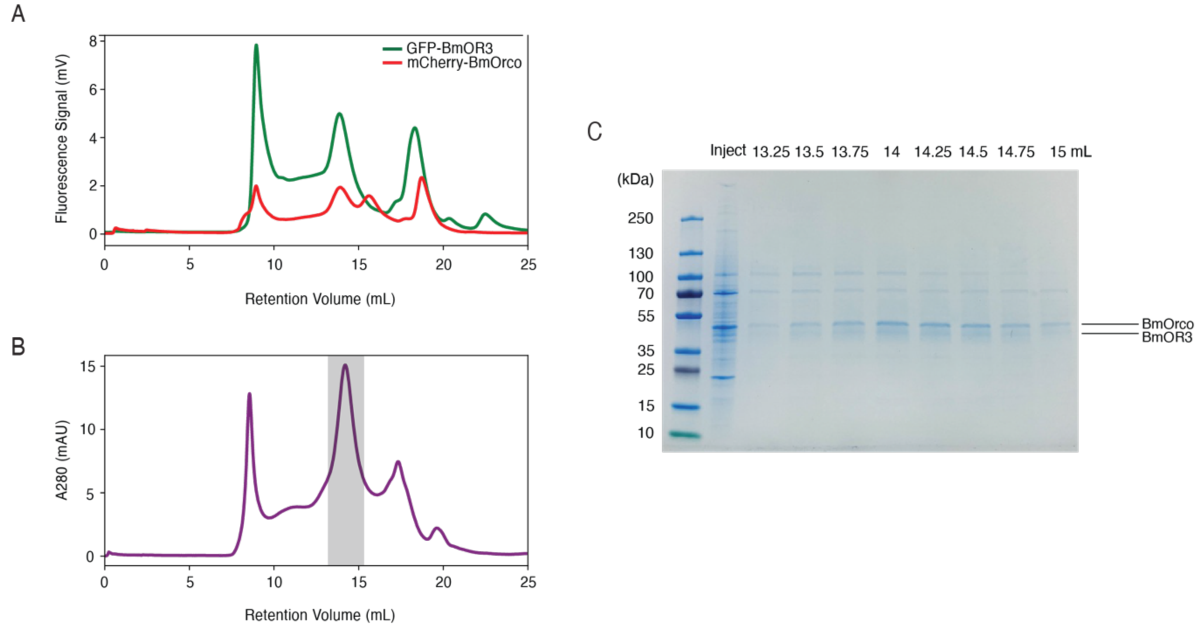

Figure S1. Purification of BmOR3/Orco complex. **(A)** Fluorescence size exclusion chromatography (FSEC) profiles of solubilized Expi293F GnTI− cells expressing GFP-BmOR3 in complex with mCherry-BmOrco. Complexes were expressed using transient transfection. **(B)** Size exclusion chromatography (SEC) profile of BmOR3/Orco purification from HEK293 cells. Fractions used for cryo-EM are highlighted in grey. **(C)** Sodium dodecyl sulfate-polyacrylamide gel electrophoresis (SDS-PAGE) of SEC run fractions. First lane, molecular weight ladder. Inject, the sample that was injected into the SEC column. Remaining lanes correspond to fractions collected at labelled retention volumes. BmOR3 and BmOrco bands are labeled.

Figure S2

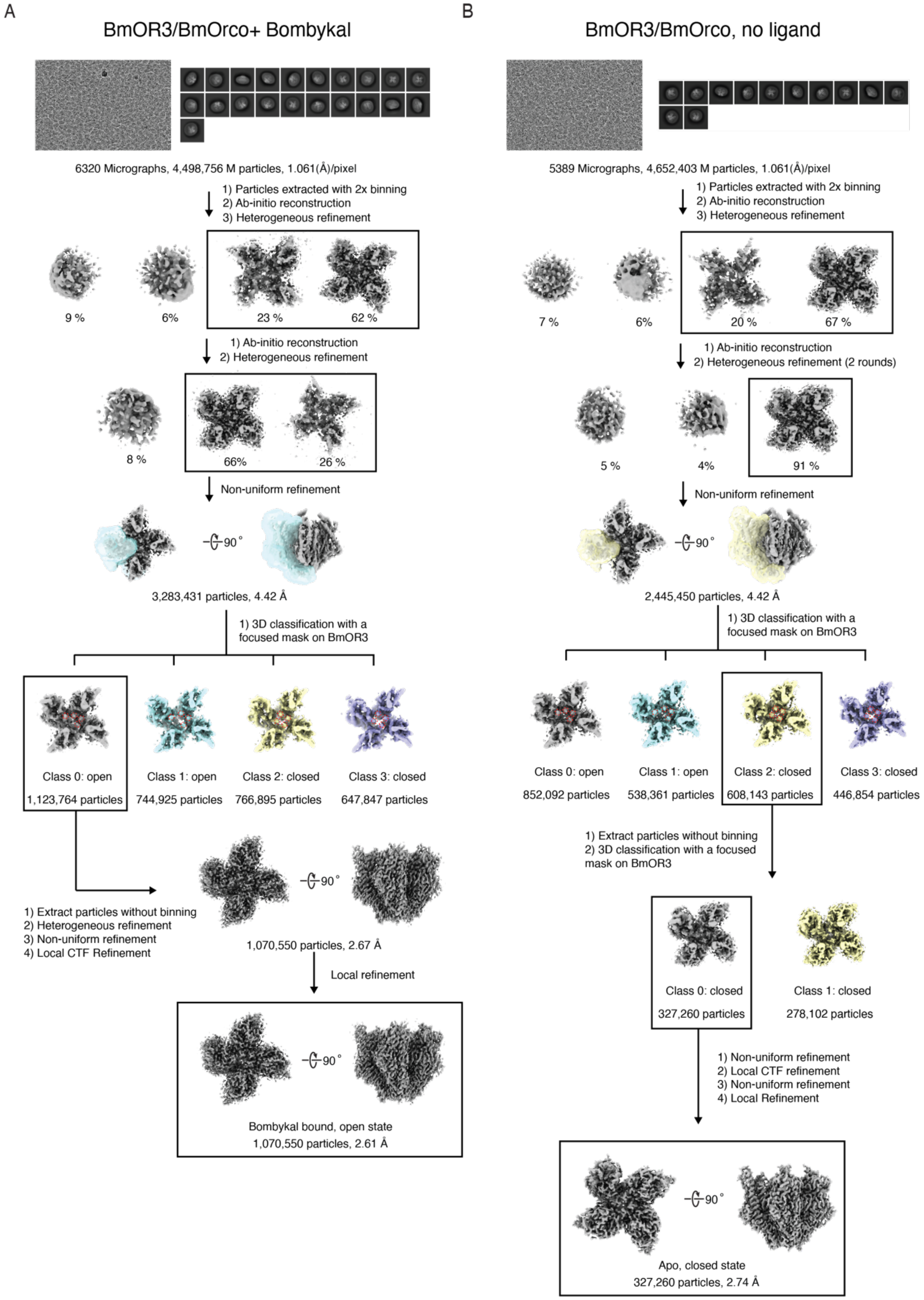
A
BmOR3/BmOrco+ Bombykal
6320 Micrographs, 4,498,756 M particles, 1.061(Å)/pixel
1) Particles extracted with 2x binning
2) Ab-initio reconstruction
3) Heterogeneous refinement
9 %
6%
23 %
62 %
1) Ab-initio reconstruction
2) Heterogeneous refinement
8 %
66%
26 %
Non-uniform refinement
90 °
3,283,431 particles, 4.42 Å
1) 3D classification with a focused mask on BmOR3
Class 0: open
1,123,764 particles
Class 1: open
744,925 particles
Class 2: closed
766,895 particles
Class 3: closed
647,847 particles
1) Extract particles without binning
2) Heterogeneous refinement
3) Non-uniform refinement
4) Local CTF Refinement
90 °
1,070,550 particles, 2.67 Å
Local refinement
90 °
Bombykal bound, open state
1,070,550 particles, 2.61 Å
B
BmOR3/BmOrco, no ligand
5389 Micrographs, 4,652,403 M particles, 1.061(Å)/pixel
1) Particles extracted with 2x binning
2) Ab-initio reconstruction
3) Heterogeneous refinement
7 %
6%
20 %
67 %
1) Ab-initio reconstruction
2) Heterogeneous refinement (2 rounds)
5 %
4%
91 %
Non-uniform refinement
90 °
2,445,450 particles, 4.42 Å
1) 3D classification with a focused mask on BmOR3
Class 0: open
852,092 particles
Class 1: open
538,361 particles
Class 2: closed
608,143 particles
Class 3: closed
446,854 particles
1) Extract particles without binning
2) 3D classification with a focused mask on BmOR3
Class 0: closed
327,260 particles
Class 1: closed
278,102 particles
1) Non-uniform refinement
2) Local CTF refinement
3) Non-uniform refinement
4) Local Refinement
90 °
Apo, closed state
327,260 particles, 2.74 Å

Figure S2. CryoEM data processing of BmOR3/Orco. Cryo-EM data processing of the BmOR3/Orco sample with (**A**) and without (**B**) bombykal.

Figure S3

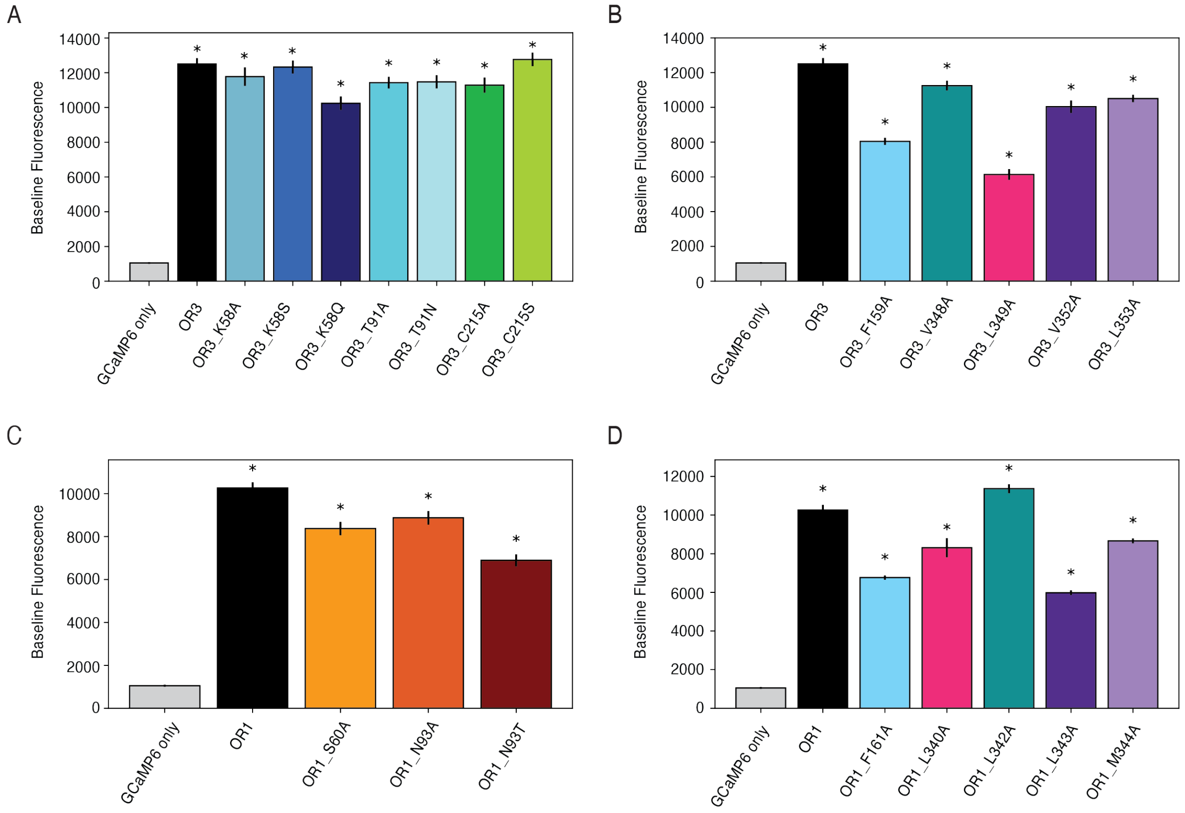

Figure S3. Baseline fluorescence of BmOR1/Orco and BmOR3/Orco mutations. (A) Baseline fluorescence of BmOR3/Orco mutants associated with the recognition domain (**B**) Baseline fluorescence of BmOR3/Orco mutants in the activation domain. (**C**) Baseline fluorescence of BmOR1/Orco mutants associated with the recognition domain. (**D**) Baseline fluorescence of BmOR1/Orco mutants in the activation domain. P-value determined using Tukey HSD (Honestly Significant Difference) comparison test of mutants against the GCaMP6 only baseline. *P < 0.05.

Figure S4

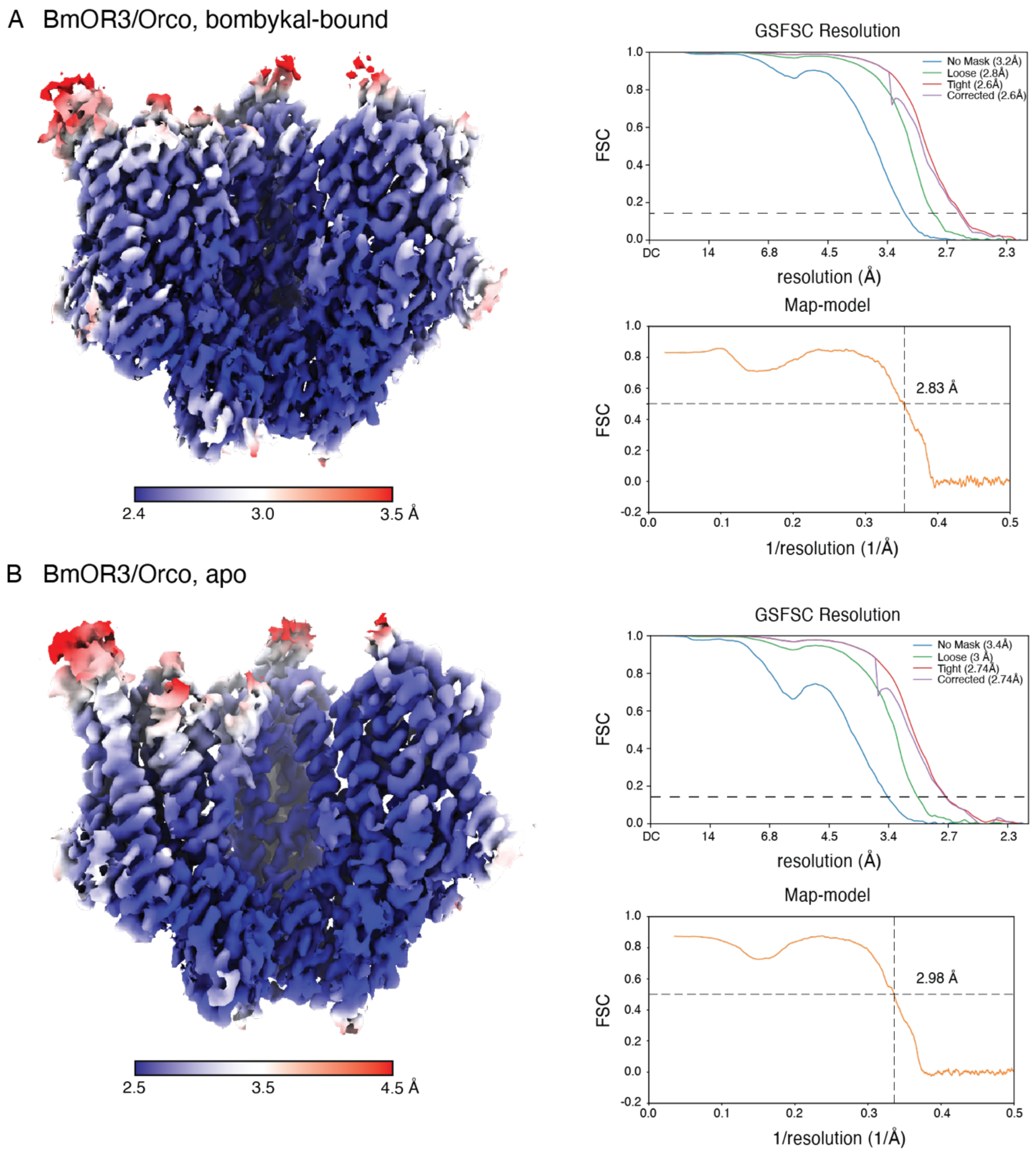

Figure S4. Summary of map quality of BmOR3/Orco samples. (**A**) Map of bombykal-bound, open state. (**B**) Map of apo, closed state. Local resolution map of bombykal bound BmOR3/Orco complex. Corresponding Gold-standard Fourier shell correlation (GS-FSC) curves are calculated in cryoSPARC. Map-model correlations are calculated in the Phenix suite. Resolutions are indicated at the FSC values denoted by the dashed line.

Figure S5

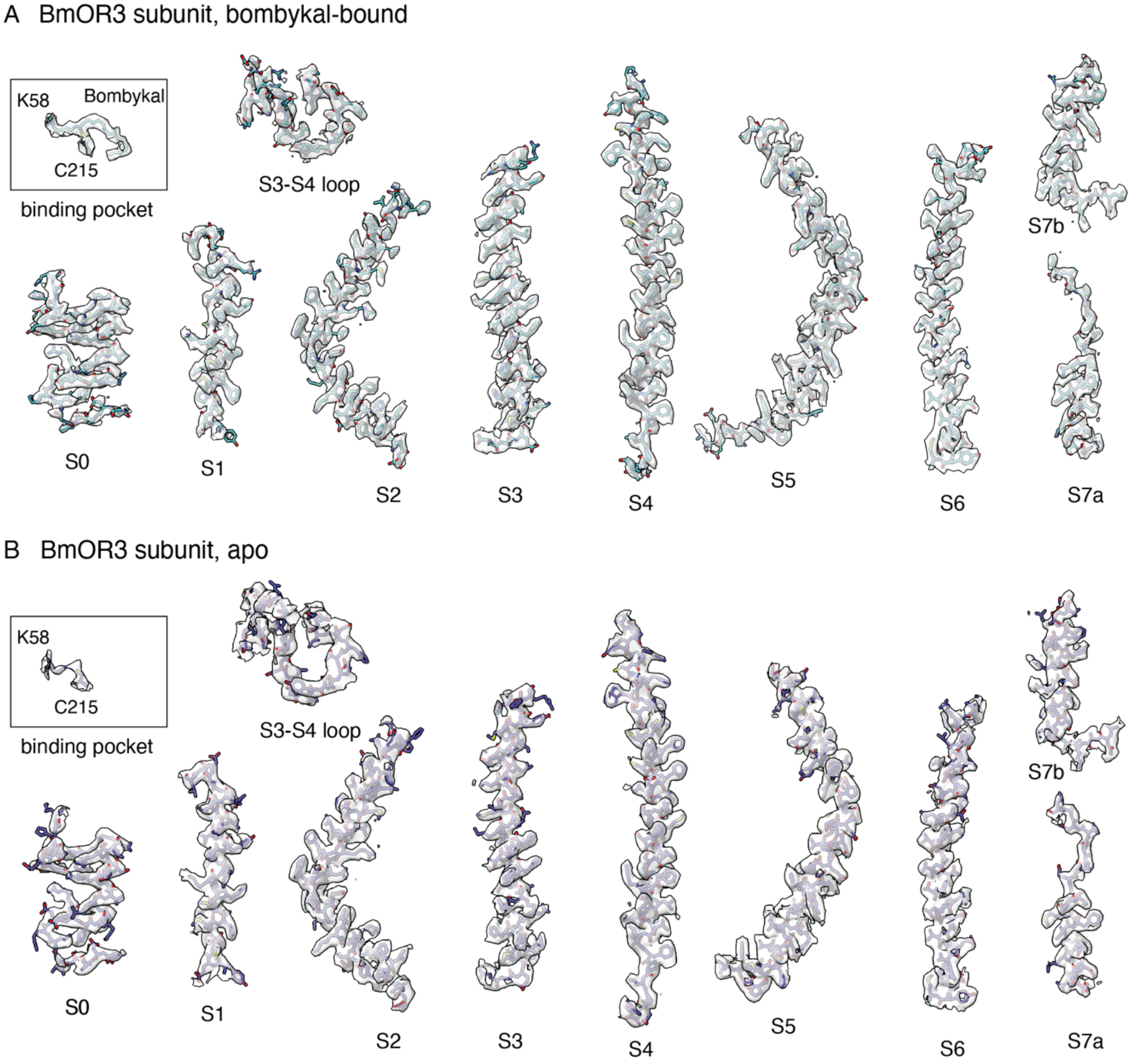

Figure S5. Details of the cryo-EM densities of BmOR3/Orco structures. Density for each segment of the BmOR3 subunit in the **(A)** bombykal-bound and the **(B)** unbound states.

Figure S6

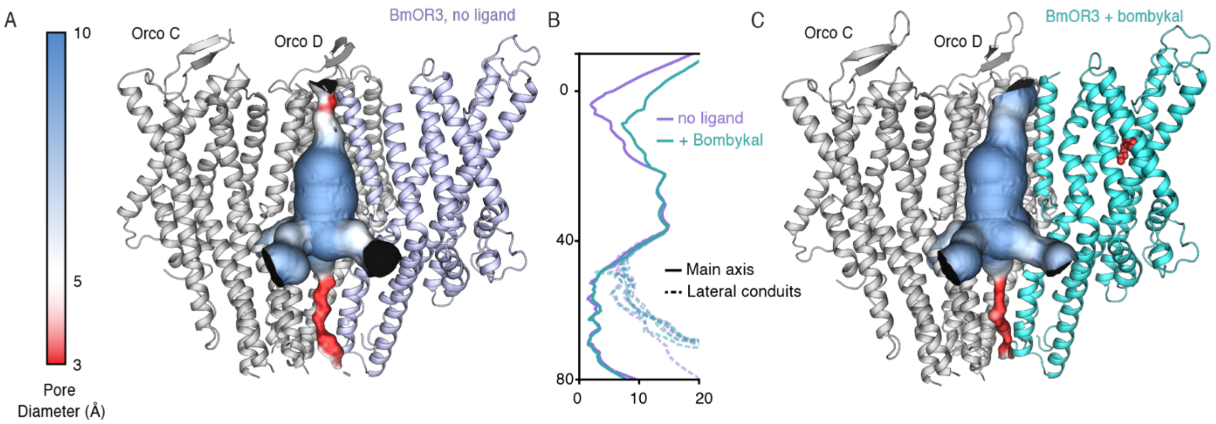

Figure S6. Details of the pore opening of the BmOR3/Orco complex. The ion permeation pathways of the unbound (**A**) and bombykal bound (**C**) structures, colored by the pore diameter. The front Orco B subunit is not shown to permit visualization of the cavity. The plot in (**B**) shows the diameter of the ion permeation pathway with its axis starting from the outer membrane boundary towards the intracellular space. The diameter of the impermeable central pathway through the anchor domain is shown in solid line, whereas those of the lateral conduits are shown in dashed lines.

Figure S7

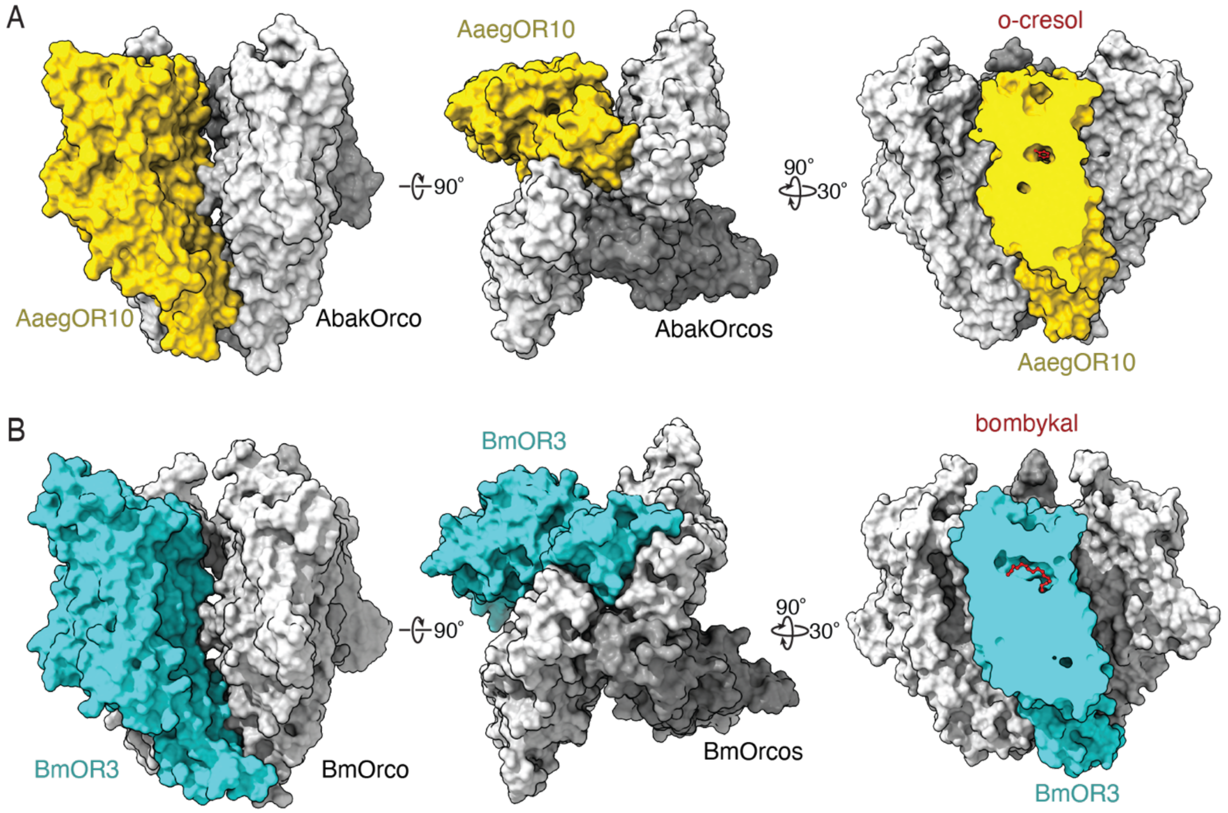

Figure S7. Comparison of AaegOR10/AbakOrco (PDB: 8v02) and BmOR3/Orco (PDB: XXXX) heteromeric complexes. (A) Left, side view of AaegOR10/AbakOrco complex. Middle, top view of the AaegOR10/AbakOrco complex. Right, slice through the AaegOR10 subunit displaying the ligand binding cavity occupied by o-cresol. **(B)** Left, side view of BmOR3/BmOrco complex. Middle, top view of the BmOR3/BmOrco complex. Right, slice through the BmOR3 subunit displaying the ligand binding cavity occupied by bombykal. BmOR3/Orco has a larger binding pocket (right images), and an extended intracellular loop that packs against a neighboring Orco subunit providing an extended intersubunit interface (middle images).

Figure S8

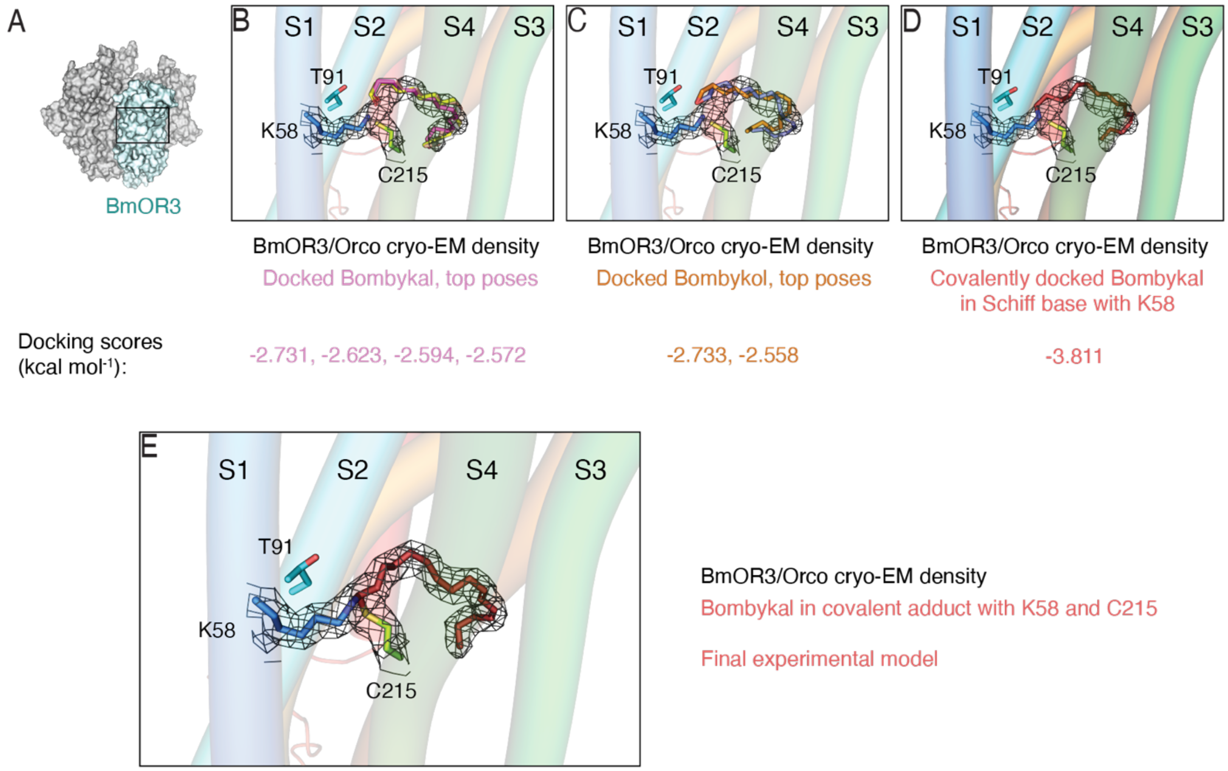

Figure S8. Bombykal cryo-EM density is best fit by covalently linking bombykal to BmOR3/Orco. (A) Surface view of the BmOR3/Orco complex, showing the orientation from which the binding pocket views are taken. (**B**) Binding pocket detail, showing the polar residues involved in interactions, the cryo-EM density for the ligand, and the top docked poses of bombykal (without covalent linkage) that best fit the density (4 poses). (**C**) Binding pocket detail in the same orientation showing the polar residues involved in interactions, the cryo-EM density for the ligand, and the top docked poses of bombykol (2 poses). (**D**) Binding pocket detail in the same orientation showing the top pose of covalently docked bombykal forming a Schiff base with K58. (**E**) Same view of the binding pocket and experimental density, with bombykal modeled as a covalent adduct involving Lys58 and Cys215.

Table S1

Construct	Replicates	Ligand	Mean (-log EC_{50} ± SE)	p-Value (-log EC_{50})	Max (ΔF/F ± SE)	p-Value (Max ΔF/F)	Hill Coefficient (n± SE)
BmOR1	9	Bombykol	6.57 ± 0.08	0.0	0.98 ± 0.07	0.974	1.6 ± 0.1
BmOR1	12	Bombykal	5.52 ± 0.07		1.02 ± 0.05		1.41 ± 0.09
BmOR3	10	Bombykol	5.89 ± 0.20	0.0	0.61±0.04	0.0	1.0 ± 0.1
BmOR3	9	Bombykal	6.79 ± 0.08		0.98±0.05		1.12 ± 0.08

Table S1. Wild-type BmOR3/Orco and BmOR1/Orco dose-response parameters. Corresponding p-values determined using Tukey HSD (Honestly Significant Difference) comparison test of BmOR1/Orco activity amongst itself and BmOR3/Orco activity amongst itself. P-values <0.0001 are rounded to 0.0 in the output of a Tukey HSD test.

Table S2

	BmOR3/BmOrco **Bombykal** (PDB: XXXX) (*EMD-XXXXX)*	BmOR3/BmOrco **Apo** (PDB: XXXX*)* *(EMD-XXXXX)*
**Data collection and Processing**		
Magnification	81,000	81,000
Voltage (kV)	300	300
Pixel size at detector (Å /pixel)	1.061	1.061
Total Electron exposure (e^-/Å^2)	50	50
Defocus range (μm)	-1 to -2	-1 to -2
Tilt angle (°)	30	30
Micrographs used	6320	5389
Total extracted particles	4,498,756	4,652,403
Final particles	1,070,550	327,260
Symmetry imposed	C1	C1
Map resolution (Å)	2.61	2.74
FSC threshold	0.143	0.143
**Refinement**		
Map sharpening B factor (Å^2)	-95	-
Model composition		
Non-hydrogen atoms	12932	12731
Protein residues	1608	1585
Ligands	1	0
R.m.s deviations		
Bond length (Å)	0.005	0.004
Bond angle (°)	0.672	0.601
Validation		
MolProbity score	1.98	1.93
MolProbity clash score	7.17	8.14
Rotamer outliers (%)	4.34	3.83
Ramachandran plot		
Favored (%)	97.5	97.8
Allowed (%)	2.5	2.2
Disallowed (%)	0	0

Table S2. Cryo-EM data collection, refinement, and validation statistics

Table S3

Construct	Replicates	Ligand	Mean (-log EC_{50} ± SE)	p-Value (-log EC_{50})	Max (ΔF/F ± SE)	p-Value (Max ΔF/F)	Hill Coefficient (n± SE)
**OR1**	**9**	**Bombykol**	**6.57 ± 0.08**	**-**	**0.98 ± 0.07**	**-**	**1.6 ± 0.1**
OR1_S60A	6	Bombykol	6.30 ± 0.08	0.9959	1.1 ± 0.1	1.0	2.1 ± 0.3
OR1_N93A	5	Bombykol	5.3 ± 0.4	0.0	0.63 ± 0.04	0.0074	1.0 ± 0.3
OR1_N93T	6	Bombykol	6.0 ± 0.1	0.106	1.02 ± 0.08	1.0	2.0 ± 0.3
OR1_L340A	5	Bombykol	5.7 ± 0.1	0.002	0.48 ± 0.08	0.0	2.0 ± 0.3
OR1_L342A	6	Bombykol	6.1 ± 0.2	0.9665	0.87 ± 0.08	0.9653	1.4 ± 0.2
OR1_I343A	5	Bombykol	-	-	0.14 ± 0.01	0.0	-
OR1_M344A	5	Bombykol	6.0 ± 0.1	0.3105	0.84 ± 0.03	0.9075	1.5 ± 0.3
OR1_F161A	6	Bombykol	-	-	0.04 ± 0.02	0.0	-
**OR3**	**10**	**Bombykol**	**5.89 ± 0.20**	**-**	**0.61 ± 0.04**	**0.0**	**1.0 ± 0.1**
OR3_K58A	4	Bombykol	6.6 ± 0.6	0.9999	0.15 ± 0.06	0.0	15 ± 6
OR3_K58S	5	Bombykol	5.5 ± 0.3	1.0	0.42 ± 0.07	0.2017	1.2 ± 0.3
OR3_K58Q	5	Bombykol	6.1 ± 0.2	1.0	0.66± 0.06	0.9993	5 ± 3
OR3_T91A	5	Bombykol	5.4 ± 0.3	0.9996	0.31 ± 0.05	0.0023	0.9 ± 0.3
OR3_T91N	5	Bombykol	6.0 ± 0.2	1.0	0.64 ± 0.07	1.0	1.7 ± 0.2
**OR3**	**9**	**Bombykal**	**6.79 ± 0.08**	**-**	**0.98 ± 0.05**	**-**	**1.12 ± 0.08**
OR3_K58A	5	Bombykal	4.8 ± 0.1	0.0	0.18± 0.03	0.0	0.8 ± 0.2
OR3_K58S	5	Bombykal	4.86 ± 0.04	0.0	0.36 ± 0.03	0.0	1.2 ± 0.2
OR3_K58Q	5	Bombykal	4.99 ± 0.08	0.0	0.68 ± 0.02	0.0003	1.3 ± 0.1
OR3_C215A	6	Bombykal	5.5 ± 0.1	0.0	0.82 ± 0.03	0.4511	1.4 ± 0.3
OR3_C215S	6	Bombykal	5.9 ± 0.1	0.0002	0.82 ± 0.05	0.3684	1.09 ± 0.07
OR3_F159A	6	Bombykal	4.9 ± 0.3	0.0	0.16 ± 0.02	0.0	4 ± 2
OR3_L349A	6	Bombykal	5.47 ± 0.06	0.0	0.48 ± 0.04	0.0	5 ± 3
OR3_L353A	6	Bombykal	6.3 ± 0.1	0.365	0.91 ± 0.04	0.9998	1.3 ± 0.2
OR3_V348A	6	Bombykal	6.32 ± 0.09	0.3911	1.13 ± 0.04	0.2269	1.25 ± 0.08
OR3_V352A	5	Bombykal	6.27 ± 0.09	0.3255	0.63 ± 0.03	0.0	1.9 ± 0.2

Table S3. BmOR1/Orco and BmOR3/Orco mutant dose-response parameters. P-values were determined using Tukey HSD (Honestly Significant Difference) comparison test against the corresponding wild-type control (bolded). Mean -log(EC_{50}) is reported for conditions in which signal saturation occurs within the tested concentration range.

References

1. Speck, K., K. Karaghiosoff, and T. Magauer, *Sequential O–H/C–H Bond Insertion of Phenols Initiated by the Gold(I)-Catalyzed Cyclization of 1-Bromo-1,5-enynes.* Organic Letters, 2015. **17**(8): p. 1982-1985.
2. Snyder, S.A., et al., *A General Strategy for the Stereocontrolled Preparation of Diverse 8- and 9-Membered Laurencia-Type Bromoethers.* Journal of the American Chemical Society, 2011. **133**(40): p. 15898-15901.
3. Cabezas, J.A. and A.C. Oehlschlager, *Stereospecific Synthesis of (E,Z)- and (Z,Z)-Hexadeca-10,12-dienal. Sex Pheromone Components of Diaphania hyalinata.* Synthesis, 1999. **1999**(01): p. 107-111.
4. Kern, D.M. and S.G. Brohawn, *Chapter Ten - SARS-CoV-2 3a expression, purification, and reconstitution into lipid nanodiscs*, in *Methods in Enzymology*, D.L. Minor and H.M. Colecraft, Editors. 2021, Academic Press. p. 207-235.
5. Punjani, A., et al., *cryoSPARC: algorithms for rapid unsupervised cryo-EM structure determination.* Nat Methods, 2017. **14**(3): p. 290-296.
6. Wang, N., et al., *Structural basis of human monocarboxylate transporter 1 inhibition by anti-cancer drug candidates.* Cell, 2021. **184**(2): p. 370-383.e13.
7. Zhao, J., et al., *Structural basis of odor sensing by insect heteromeric odorant receptors.* Science, 2024. **384**(6703): p. 1460-1467.
8. Terwilliger, T.C., et al., *Improvement of cryo-EM maps by density modification.* Nature Methods, 2020. **17**(9): p. 923-927.
9. Jumper, J., et al., *Highly accurate protein structure prediction with AlphaFold.* Nature, 2021. **596**(7873): p. 583-589.
10. Varadi, M., et al., *AlphaFold Protein Structure Database in 2024: providing structure coverage for over 214 million protein sequences.* Nucleic Acids Res, 2024. **52**(D1): p. D368-D375.
11. Emsley, P., et al., *Features and development of Coot.* Acta Crystallogr D Biol Crystallogr, 2010. **66**(Pt 4): p. 486-501.
12. Adams, P.D., et al., *PHENIX: a comprehensive Python-based system for macromolecular structure solution.* Acta Crystallogr D Biol Crystallogr, 2010. **66**(Pt 2): p. 213-21.
13. Morin, A., et al., *Collaboration gets the most out of software.* eLife, 2013. **2**: p. e01456.
14. Halgren, T.A., et al., *Glide: A New Approach for Rapid, Accurate Docking and Scoring. 2. Enrichment Factors in Database Screening.* Journal of Medicinal Chemistry, 2004. **47**(7): p. 1750-1759.
15. Zhu, K., et al., *Docking Covalent Inhibitors: A Parameter Free Approach To Pose Prediction and Scoring.* Journal of Chemical Information and Modeling, 2014. **54**(7): p. 1932-1940.
16. Smart, O.S., et al., *HOLE: A program for the analysis of the pore dimensions of ion channel structural models.* Journal of Molecular Graphics, 1996. **14**(6): p. 354-360.
17. Butterwick, J.A., et al., *Cryo-EM structure of the insect olfactory receptor Orco.* Nature, 2018. **560**(7719): p. 447-452.
18. Del Marmol, J., M.A. Yedlin, and V. Ruta, *The structural basis of odorant recognition in insect olfactory receptors.* Nature, 2021. **597**(7874): p. 126-131.

19. Yang, C., et al., *Sex Pheromone Receptors of Lepidopteran Insects.* Frontiers in Ecology and Evolution, 2022. **Volume 10 - 2022**.
20. Fu, L., et al., *CD-HIT: accelerated for clustering the next-generation sequencing data.* Bioinformatics, 2012. **28**(23): p. 3150-2.
21. Gilchrist, C.L.M., M. Mirdita, and M. Steinegger, *Multiple Protein Structure Alignment at Scale with FoldMason.* bioRxiv, 2024: p. 2024.08.01.606130.
22. Minh, B.Q., et al., *IQ-TREE 2: New Models and Efficient Methods for Phylogenetic Inference in the Genomic Era.* Molecular Biology and Evolution, 2020. **37**(5): p. 1530-1534.
23. Ciccarelli, F.D., et al., *Toward automatic reconstruction of a highly resolved tree of life.* Science, 2006. **311**(5765): p. 1283-7.
